# Association of layer-specific knee cartilage T2-relaxation measurements with age, sex and cartilage morphology at 1.5-T MRI

**DOI:** 10.1007/s00330-025-11806-8

**Published:** 2025-07-25

**Authors:** Katharina Aschauer, Marc-André Weber, Robin Bülow, Norbert Hosten, Matthias Seitel, Carsten Oliver Schmidt, Hanjo Marquardt, Frank Weber, Bastian Klaan

**Affiliations:** 1 Institute of Diagnostic and Interventional Radiology, Pediatric Radiology and Neuroradiology, University Medicine Rostock, Rostock, Germany; 2 Institute of Diagnostic Radiology and Neuroradiology, University Medicine Greifswald, Greifswald, Germany; 3 Mint Medical GmbH, Heidelberg, Germany; 4 Institute for Community Medicine, SHIP/Clinical-Epidemiological Research, University Medicine Greifswald, Greifswald, Germany; 5 Institute for Biostatistics and Informatics in Medicine and Ageing Research, University Medicine Rostock, Rostock, Germany

**Keywords:** Magnetic resonance imaging, Osteoarthritis, Cartilage, articular, Relaxation time, Knee joint

## Abstract

**Objective:**

This study aimed to establish normal knee cartilage T2-values at 1.5-Tesla, assess the influence of age and sex on T2-values, and compares T2-times between subjects with and without morphological cartilage changes.

**Materials and methods:**

A sagittal 2D T2-weighted multi-slice multi-echo sequence (MSME) sequence with automatic generation of a color-coded T2-map was acquired at 1.5-Tesla in 929 volunteers (ages 28–89) from the Study-of-Health-in-Pomerania TREND-1 cohort. Knee morphology was assessed with the modified Noyes Score in eight cartilage regions. T2 measurements were performed manually in seven cartilage regions, including superficial and deep cartilage layers.

**Results:**

Subjects with normal cartilage morphology (300 subjects) showed significant T2-value differences across cartilage regions (*p* ≤ 0.001), with higher values in femoral cartilage and superficial layers. T2-values increased with age (*p* ≤ 0.001), and women had higher T2-values in the femoral, tibial, and medial femorotibial compartments. The subjects with evidence of pathological cartilage morphology changes (629 subjects) had higher T2-values compared to the subjects with structurally normal knee cartilage in MRI (*p* ≤ 0.001).

**Conclusions:**

This study provides population-based 1.5-Tesla knee cartilage T2-values, showing age-related increases and higher values in superficial and femoral layers. Pathological cartilage morphology was associated with elevated T2-values.

**Key Points:**

***Question*** *This study examines early cartilage degeneration by establishing normal T2-values and analyzing how demographics and morphological cartilage changes impact these values*.

***Findings*** *T2-times were higher in superficial femoral cartilage but lower in retropatellar, tibial cartilage, and deep layers, increasing with age and pathological cartilage changes*.

***Clinical relevance*** *This study establishes normal T2-values for knee cartilage at 1.5-Tesla, identifies age- and sex-related variations, and associates elevated T2-values to morphological cartilage changes, enhancing cartilage health understanding and early diagnostic precision*.

**Graphical Abstract:**

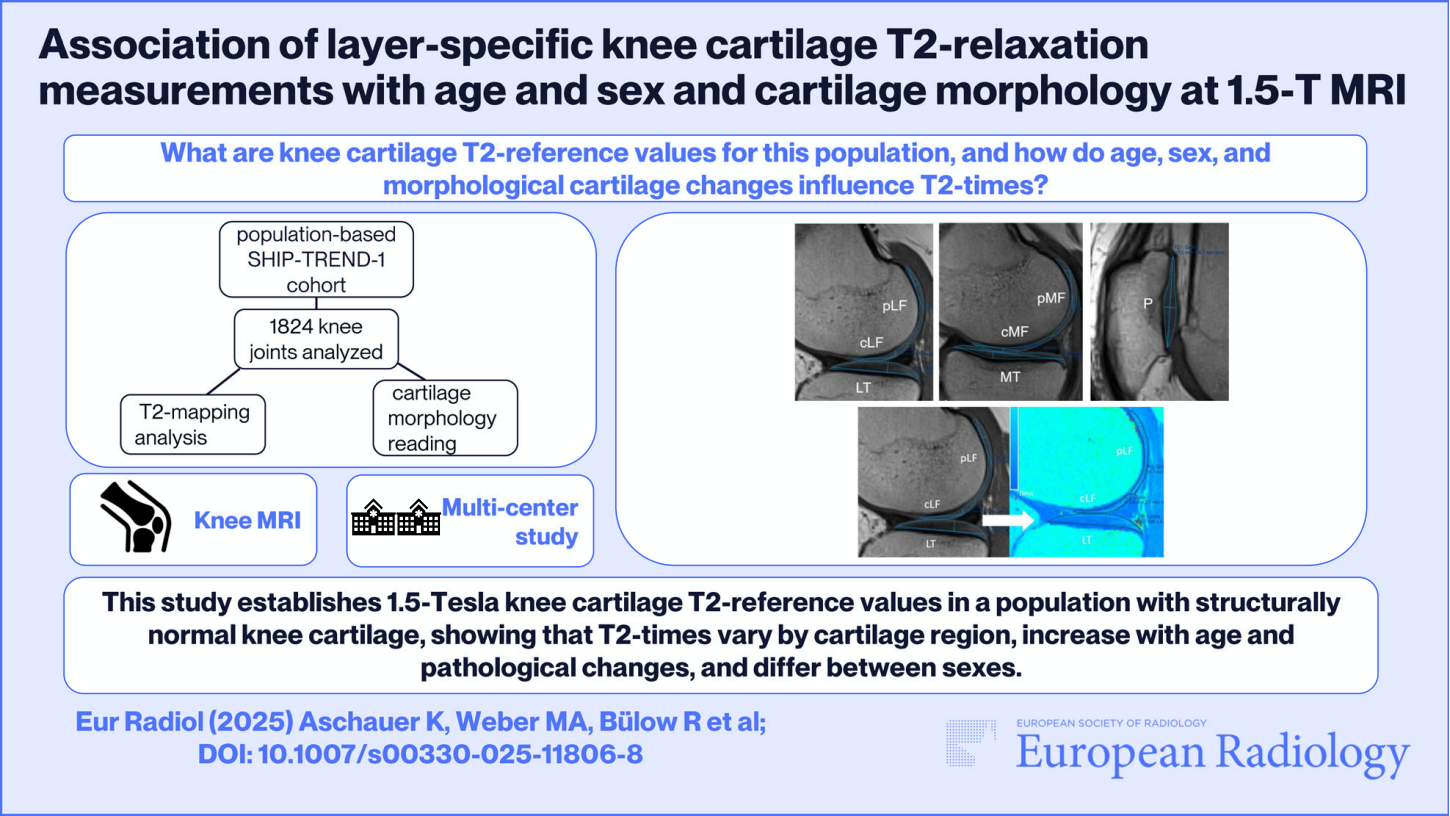

## Introduction

Osteoarthritis (OA) is the most common adult joint disease with significant socioeconomic impact [1, 2], yet its pathophysiology remains incompletely understood [3]. While morphologic magnetic resonance imaging (MRI) is the gold standard for visualizing cartilage defects and internal changes with high resolution, it often detects changes that are already irreversible. Before these morphologically detectable degenerative lesions appear, biochemical changes in the cartilage occur [4, 5].

MRI-derived cartilage spin-spin (transverse) relaxation times (T2) are sensitive to early biochemical changes, such as proteoglycan loss, collagen network fragmentation, and increased water content, which are indicative of early cartilage degeneration [6, 7]. Thus, T2-relaxation time is a promising non-invasive biomarker for early OA stages [6–8]. In healthy individuals, cartilage T2-relaxation time varies between superficial and deep cartilage layers (from cartilage surface to bone interface) [9, 10] and between different knee cartilage plates [11, 12]. T2 measures can distinguish between healthy cartilage and that of individuals with or at risk for knee OA [13, 14]. Furthermore, T2-values correlate with OA risk factors like age [11, 12, 15, 16], obesity [11, 17, 18], physical activity [19–21], and cartilage lesions [8, 22].

Although female gender is a known risk factor for OA [23–25], studies on its relationship with T2-values have been inconclusive [9, 11, 12, 26, 27]. Establishing reference T2-values for knee cartilage can enhance understanding of OA pathophysiology and facilitate early detection and severity estimation, similar to bone mineral density reference data in osteoporosis. Previous studies have explored knee cartilage T2-relaxation times in healthy subjects [10–12, 28], but not at the commonly used 1.5-T MRI. Additionally, these studies often examined “bulk” cartilage without differentiating between regions and layers, which show significant differences in T2-relaxation times [10–12].

Our study aimed to provide MRI-based T2-relaxation time reference data for layer- and subregion-specific cartilage composition in an adult cohort without MRI-based evidence of pathological cartilage changes (modified Noyes Score 0 [29, 30]) and without inlying surgery material or other evidence of prior knee surgery. We differentiated between superficial and deep layers, medial and lateral compartments, as well as weight-bearing and non-weight-bearing cartilage subregions. We also examined the relationships between layer- and region-specific T2-times and population-based data, and compared T2-relaxation times between subjects with and those without pathological changes in the knee cartilage morphology in MRI.

## Materials and methods

### Study participants

The Study-of-Health-in-Pomerania (SHIP; http://www2.medizin.uni-greifswald.de/cm/fv/ship.html) is an ongoing longitudinal population-based cohort study designed to assess the prevalence and incidence of risk factors and diseases in north-eastern Germany. SHIP consists of three cohorts (SHIP-START, SHIP-TREND, and SHIP-NEXT) and includes clinical-epidemiological data and whole-body MRI [31]. Representative samples were drawn from population registries for each cohort. The study was approved by the institutional review board, and informed consent was obtained from each participant [31, 32]. The manuscript was written according to the STROBE criteria [33].

SHIP-TREND-0 comprised 4420 participants with a subsample of 2186 subjects with whole-body-MRI. After a follow-up time of over 5 years, the SHIP-TREND-1 cohort was performed with 1497 whole-body-MRI and a subgroup of 929 volunteers with MRI of knee joints in SHIP-TREND-1.

A total of 913 subjects (*n* = 913/929) received images of both knee joints; in the remaining 16 subjects, only one knee joint was examined due to prior arthroplasty. For the underlying image analysis, we retrospectively characterized subgroups of knees with morphological normal knee cartilage in MRI and with morphological cartilage changes.

In total, 1842 knee joints were evaluated morphologically, but 18 joints were excluded from T2 analysis due to unavailable T2-maps (*N* = 9) or pronounced movement artifacts (*N* = 9). Ultimately, 1824 knee joints (924 subjects) met all requirements for inclusion in the T2 analysis. A cohort with morphological normal knee cartilage (*N* = 300) and a cohort with morphological cartilage changes in MRI (*N* = 624) were defined for T2-mapping analysis. The cohort with structurally normal cartilage included subjects with normal cartilage morphology in both knees (modified Noyes Score = 0 in all cartilage regions), no MRI-based evidence of prior knee surgery like ligament reconstruction or visible surgery material and no evidence of obvious posttraumatic changes like healed fractures with bone deformity. The cohort with pathological changes in the knee joint included individuals who did not meet these criteria.

### MR imaging

All examinations were performed at the same 1.5-T MR scanner (Magnetom Avanto; Siemens Healthineers) using a Tx/Rx 15-channel knee coil. Subjects were scanned in a supine position with the patellar articular surface parallel to B0 and the weight-bearing femoral and tibial cartilage articular surface perpendicular to B0. The following sequences were used for morphologic knee analysis (including modified Noyes Scoring [29, 30]): sagittal 3D proton-density weighted fat-saturated fast-spin-echo sequence (SPACE) with coronal and axial reformation, axial 2D T1-weighted spin-echo (SE) sequence, and coronal 2D T1-weighted SE sequence. A sagittal 2D T2-weighted MSME with automatic inline calculation of parametric, color-coded T2-maps by the built-in MapIt (version 1.0 Siemens Healthineers) was used for cartilage T2 measurements. Data processing method of MapIt comprised a pixel-wise, mono-exponential, non-negative least-square fit analysis, omitting the value for the first echo to reduce error resulting from signals produced by the stimulated echo. The sagittal MSME sequence for quantitative T2-mapping was performed with a TR of 1060 ms, TEs of 13.8 ms, 27.6 ms, 41.4 ms, 55.2 ms, and 69 ms, and a flip-angle of 180°. The FOV was 160 × 160 mm, the pixel matrix was 256 × 256, and the slice thickness was 3.0 mm, resulting in a voxel size of 0.6 × 0.6 × 3.0 mm. The bandwidth was 227 Hz/Px, and the data acquisition time for this sequence was 2:23 min for 14 slides. All other sequence parameters are provided in a table in the supplementary material.

### Image analysis

**Fig. 1:**
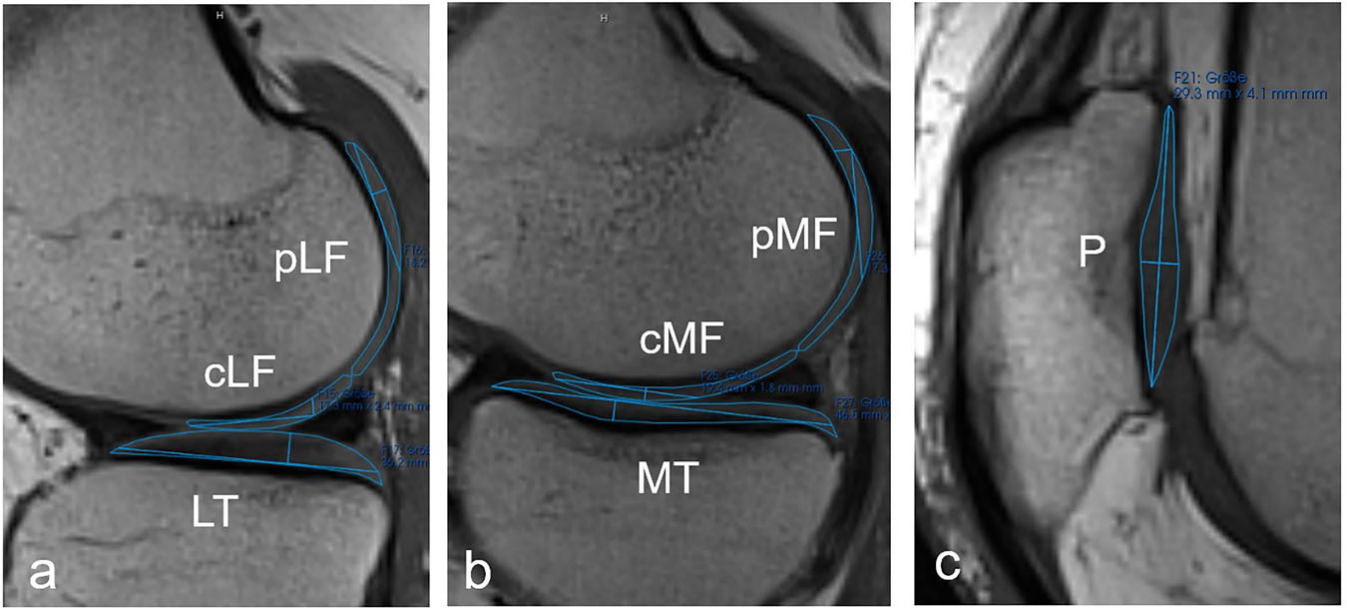
Sagittal multi-slice multi-echo spin-echo sequence (MSME-SE) with the lowest acquired echo time (13.8 ms) showing the defined joint compartments. Additionally plotted long and short axis within the ROI (blue lines) for automatic calculation of the ROI area. **a** Central and posterior lateral femoral condyle (cLF, pLF), lateral tibia (LT). **b** Central and posterior medial femoral condyle (cMF, pMF), medial tibia (MT). **c** Retropatellar cartilage (P). Source: study participant from the SHIP-TREND-1 data pool

**Fig. 2:**
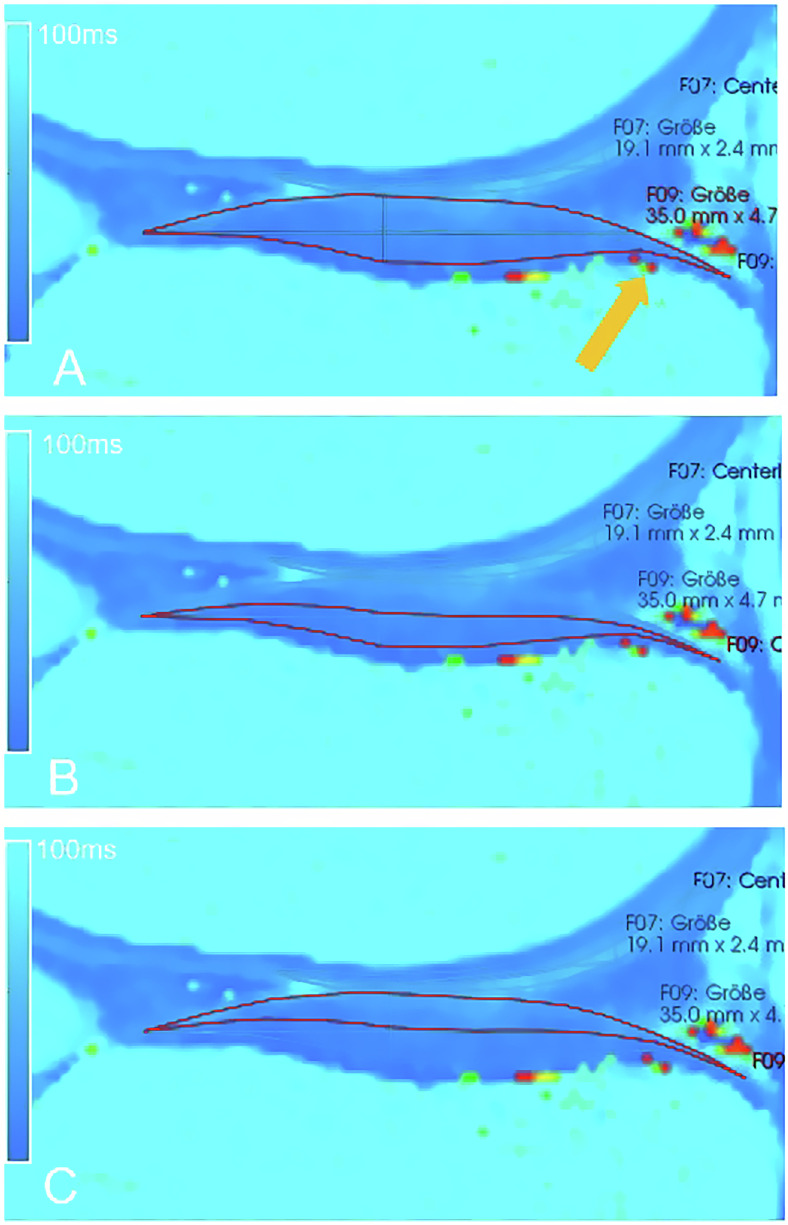
Sagittal T2-map MSME-SE sequence in rainbow color scheme showing manually created total ROI at lateral tibia (**A**, outlined in red) and program function generated subdivision into deep (**B**) and superficial cartilage layer (**C**) (outlined in red). Chemical artifact at the bone-cartilage interface with single pixels of high intensity (arrow in **A**). Source: study participant from the SHIP-TREND-1 data pool

Cartilage T2-mapping analysis and morphological knee reading (including modified Noyes Grading) were performed using proprietary software mint Lesion™ (Mint Medical GmbH). Two experienced readers (K.A. and H.M.) independently performed cartilage segmentation, with quality control and/or correction by an expert reader with over 10 years of experience in musculoskeletal radiology (B.K.). Regions of interest (ROIs) were manually drawn to delineate the cartilage in seven regions: patella (P), medial and lateral tibia (MT/LT), medial and lateral weight-bearing femoral condyles (cMF/cLF), and medial and lateral posterior femoral condyles (pMF/pLF). The tibial cartilage was segmented from anterior to posterior, while the central weight-bearing compartment of the femoral cartilage was defined as the area between the posterior edge of the anterior meniscus and the posterior edge of the posterior meniscus (Fig. 1). ROIs were manually drawn into the T2 MSME sequence and then copied into the color-coded T2-map at the same anatomical position. Segmentation could not be done in some cartilage regions (*N* = 259) due to artifacts or complete cartilage depletion (Noyes grade 4). Each segmented cartilage region was automatically divided into a superficial and deep layer, each representing 50% of the distance between the segmented cartilage surface and the bone interface (Fig. 2). For each region, we analyzed the whole, the superficial and the deep cartilage. T2 maps were computed on a pixel-by-pixel basis using all five echoes (TE = 13.8–69.0 ms). By averaging the T2-values of all compartments analyzed, we calculated the mean T2-time for the entire knee joint. The same approach was used to calculate the mean T2-time of the regions in the medial compartment (MC = MT, cMF, pMF), the lateral compartment (LC = LT, cLF, pLF), the femur (F = cMF, pMF, cLF, pLF), and the tibia (T = LT, MT).

The analysis of the knee cartilage included rating morphology in eight knee regions (medial and lateral central femur, medial and lateral posterior femur, medial and lateral tibia, patella, trochlea) using modified Noyes score [30, 34]. For this analysis, all available morphological MR sequences were used (3D SPACE, 2D T1w SE).

Interobserver variability of T2-mapping analysis and modified Noyes Score was calculated in a random sample of 100 knee MRI datasets. Intraobserver reproducibility was obtained by re-evaluating 40 randomly selected cases after at least 4 weeks, blinded to the previous measurement. Both inter- and intrarate reproducibility was performed by all three readers.

### Statistical analysis

Statistical analyses were conducted using IBM SPSS 27 software. Baseline T2-values were described using mean, standard deviation, and 95% confidence intervals. Intra- and interrater reproducibility of T2 analysis was calculated using the intraclass correlation coefficient (ICC) and the root mean square average coefficients of variation (RMSA CV [35]), as was done in previous studies [36, 37]. Cohen’s kappa coefficient was used to assess the reliability of morphological cartilage evaluation.

A one-factorial analysis of variance with post hoc tests was used to investigate the influence of cartilage layers and regions on T2-time in subjects with morphological normal knee cartilage based on MRI. T-tests were used for regional differences between medial and lateral compartments, as well as for differences between women and men and between the two defined cohorts. Age differences were analyzed by assigning participants with morphological normal knee cartilage to one of four age groups according to age quartiles. A one-factorial analysis of variance with post hoc tests, correlation analysis (Pearson), and regression analysis was used to investigate age influence on T2-times. A mixed linear regression was calculated to account for multiple T2 measurements per subject, considering sex, age, cartilage region, and cohort membership (morphological normal knee cartilage versus pathological changes in knee cartilage) as influencing factors. Statistical significance was defined as *p*-values < 0.05, with Bonferroni correction applied for multiple testing.

## Results

### Subject characteristics

The study population included 929 subjects aged 28–89 years (mean age = 56.89 years, SD = 12.73 years) and is presented in Table 1. The T2 analysis covered 924 subjects, split evenly between women (49.9%) and men (50.1%). The cohort with morphological normal knee cartilage had 300 subjects (48.3% women, 51.7% men) aged 28–85 years, and the cohort with pathological cartilage changes had 624 subjects (50.6% women, 49.4% men) aged 29–89 years. No significant differences in sex distribution were found; however, the cohort with evidence of structural cartilage changes was significantly older than the other cohort by an average of 7.998 years (t = −9.320; *p* < 0.001; *n* = 918).

**Table 1:** Demographic characteristics

	All participants (*n* = 929)	Normal cartilage morphology cohort (*n* = 300)	Abnormal cartilage morphology cohort (*n* = 629)	T2-mapping (*n* = 924)
Age (M ± SD)	56.89 ± 12.73	51.51 ± 12.94	59.48 ± 11.78	56.89 ± 12.73
Female (*N*/%)	462 (49.7%)	145 (48.3%)	319 (50.7%)	461 (49.9%)
Male (*N*/%)	467 (50.3%)	155 (51.7%)	310 (49.3%)	463 (50.1%)

### Reproducibility

Interobserver reproducibility for T2 analysis demonstrated ICCs ranging from 0.80 to 0.96 and RMS CVs between 5 and 12%. Intraobserver reproducibility showed ICCs of 0.96 to 1.00 and RMS CVs of 2 to 7%. Modified Noyes Score analysis achieved interobserver agreement rates of 84.6 to 99.8% and intraobserver agreement rates of 93.3 to 99.7%, with Cohen’s κ values of 0.85 and 0.97, respectively.

### Knee cartilage T2-relaxation time in subjects with morphological normal knee cartilage

**Fig. 3:**
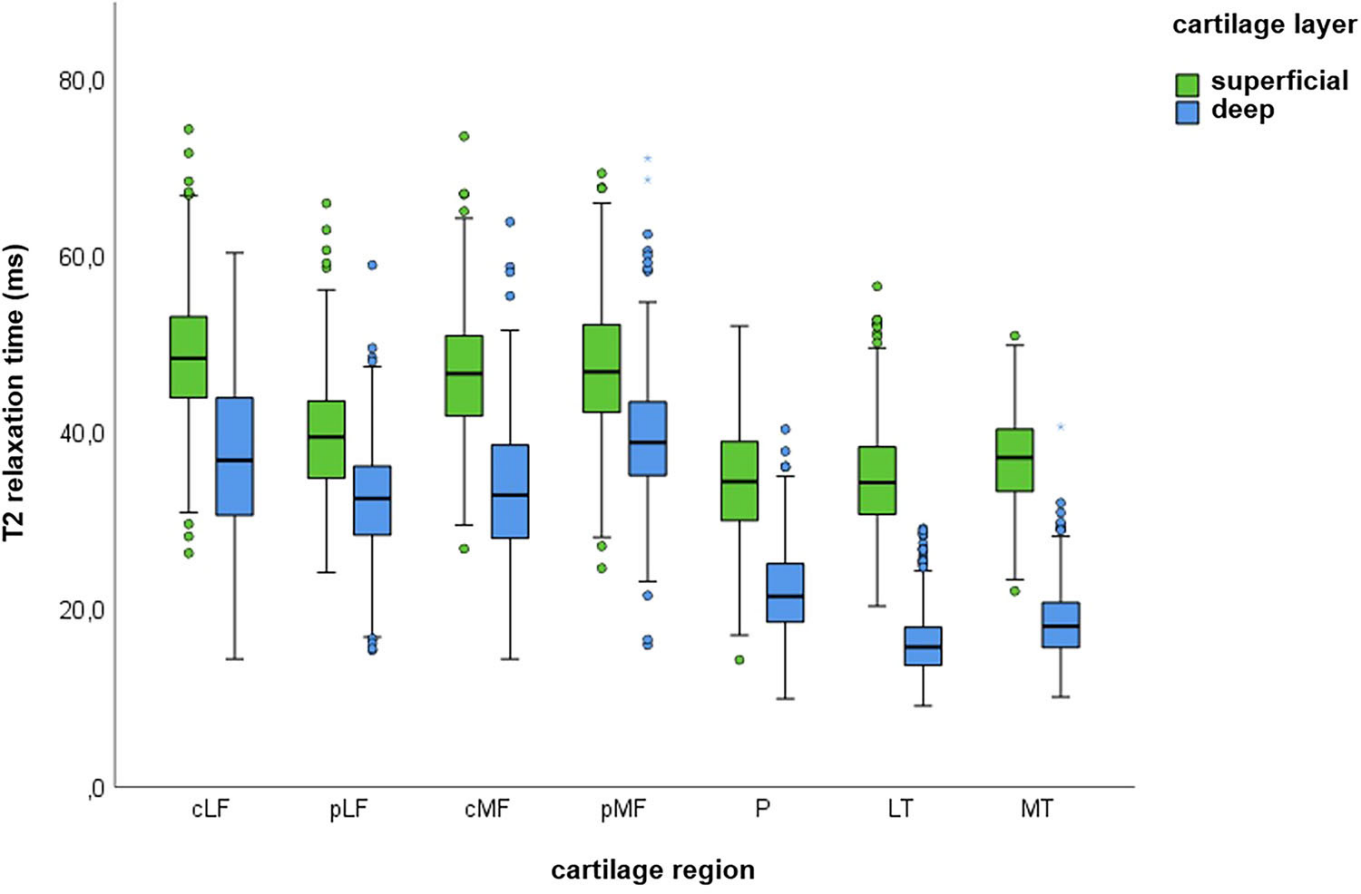
T2-relaxation times of the superficial and deep cartilage layers of subjects with normal knee cartilage morphology. The T2-times of the cartilage layers differed significantly in all regions (*p* = < 0.001)

The overall mean T2-time for knee cartilage in these subjects was 35.10 ms (SD = 8.99 ms). The superficial cartilage layer (mean T2 = 41.31 ms, SD = 8.72 ms) had significantly higher T2-times compared to the deep cartilage layer (mean T2 = 28.55 ms, SD = 10.73 ms) (t(7808.184) = 58.87, *p* < 0.001). A mean difference of 12.76 ms between the superficial and deep T2-times was noted for the entire knee joint (Table 2, Fig. 3).

**Table 2:** T2-relaxation times (in ms) of the cohort with normal cartilage morphology in MRI divided into cartilage regions and layers

Region	*N*	M	SD	Min	Max
Total w	4069	35.10	8.99	16.30	69.50
Total sf	4069	41.31	8.72	14.40	74.50
Total dp	4069	28.55	10.73	9.20	71.20
cLF w	580	43.35	6.76	26.60	62.10
cLF sf	580	48.94	7.08	26.50	74.50
cLF dp	580	37.60	8.96	14.50	60.50
pLF w	572	36.17	5.66	19.90	62.70
pLF sf	572	39.57	6.46	24.30	66.10
pLF dp	572	32.53	6.06	15.50	59.10
cMF w	577	40.32	6.68	22.90	69.50
cMF sf	577	46.84	7.01	27.00	73.70
cMF dp	577	33.54	7.86	14.50	64.00
pMF w	575	43.58	6.49	20.30	66.30
pMF sf	575	47.32	7.71	24.80	69.50
pMF dp	575	39.55	6.75	16.10	71.20
P w	591	28.59	5.05	16.30	42.90
P sf	591	34.75	6.40	14.40	52.20
P dp	591	22.17	4.87	10.00	40.50
LT w	588	25.85	4.08	16.70	39.30
LT sf	588	34.95	5.93	20.50	56.70
LT dp	588	16.34	3.55	9.20	29.30
MT w	586	28.28	3.94	17.20	42.30
MT sf	586	37.14	5.18	22.20	51.10
MT dp	586	18.70	3.90	10.20	40.80

Total: all cartilage regions of the knee

*cLF/pLF* central/posterior lateral femoral condyle, *cMF/pMF* central/posterior medial femoral condyle, *P* retropatellar cartilage, *LT* lateral tibia, *MT* medial tibia, *w* whole, *sf* superficial, *dp* deep

Additionally, significant differences were found across various cartilage regions (*p* < 0.001), though not all pairwise comparisons showed significant differences after Bonferroni correction.

**Fig. 4:**
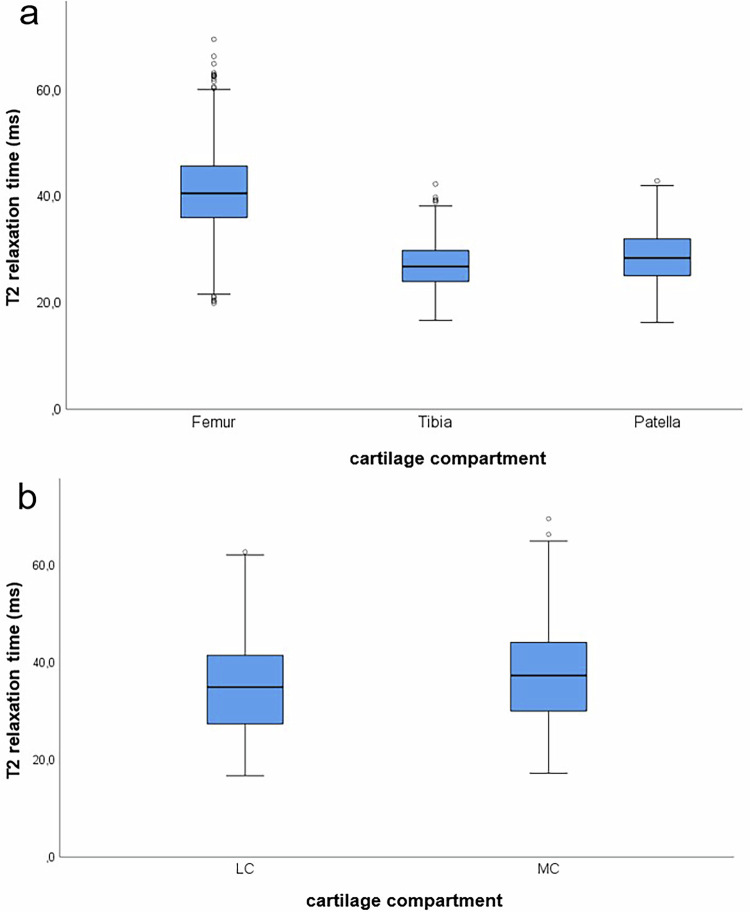
Illustration of the T2-relaxation time of the knee joint cartilage of subjects with normal knee cartilage morphology, divided into additionally defined compartments. **a** T2-relaxation times of the femoral, tibial and patellar cartilage. **b** T2-relaxation times of the lateral (LC) and medial compartments (MC) (Note: Femur = cLF, pLF, cMF, pMF; Tibia = LT, MT; LC = cLF, pLF, LT; MC = cMF, pMF, MT)

The T2-values of different knee compartments also showed significant differences: the femur had higher T2-times (mean = 40.87 ms, SD = 7.08 ms) than the tibia (mean = 27.06 ms, SD = 4.19 ms) and patella (mean = 28.59 ms, SD = 5.05 ms) (*p* < 0.001). Pairwise comparison indicated that patellar cartilage had higher T2-values than tibial cartilage (mean difference = −1.53 ms, *p* < 0.001) (Fig. 4a). Furthermore, T2-values were higher in the medial compartment (mean = 37.34 ms, SD = 9.13 ms) compared to the lateral compartment (mean = 35.07, SD = 8.80 ms) (t(3476) = −7.442, *p* < 0.001) (Fig. 4b).

### Reference values for cartilage T2-times

Due to significant differences between the cohorts, as well as age- and sex-specific variations, reference values were calculated for subjects with morphological normal cartilage based on MRI by cartilage region (Table 3). T2-values increased by 15–20 ms from the 5th to the 95th percentile, with women showing an increase of 18.46 ms and men of 17.19 ms. Age-specific reference values indicated a mean increase of 3 ms in T2-time from the youngest to the oldest age group (Table 4).

**Table 3:** Sex-specific measurements of the percentiles of the T2-relaxation time (in ms) of the knee joint cartilage of subjects with normal cartilage morphology in MRI by cartilage region

	Region	*N*	5%	10%	25%	50%	75%	90%	95%
Male	cLF	302	32.60	34.03	37.20	41.60	46.20	50.68	52.80
	pLF	299	28.40	30.30	33.40	37.30	40.70	44.30	45.70
	cMF	292	29.20	30.93	35.25	39.60	44.25	49.31	51.30
	pMF	291	34.20	35.72	38.80	42.70	47.00	50.68	52.60
	P	306	21.00	22.94	25.90	28.90	32.40	35.99	38.60
	LT	304	20.40	21.30	23.00	25.25	27.70	30.45	32.30
	MT	304	22.00	23.20	25.05	27.80	30.60	33.20	34.80
Female	cLF	278	34.10	36.09	39.90	45.15	49.40	53.23	56.30
	pLF	273	26.20	28.22	31.40	35.20	38.60	41.58	44.50
	cMF	285	30.30	32.72	36.20	40.80	45.20	48.90	51.60
	pMF	284	32.50	35.25	39.75	44.50	48.90	52.50	54.80
	P	285	19.50	22.04	24.30	27.50	31.50	34.64	36.20
	LT	284	19.50	20.65	22.95	25.85	29.00	32.10	34.70
	MT	282	22.10	23.30	26.00	28.65	31.50	33.67	35.30

*cLF/pLF* central/posterior lateral femoral condyle, *cMF/pMF* central/posterior medial femoral condyle, *P* retropatellar cartilage, *LT* lateral tibia, *MT* medial tibia

**Table 4:** Age- and sex-specific measurements of the percentiles of the T2-relaxation time (in ms) of the knee joint cartilage of subjects with normal cartilage morphology in MRI by cartilage region

	Region	*N*	5%	10%	25%	50%	75%	90%	95%
**Age group 1 (28–41 years)**									
Male	cLF	78	30.89	32.69	35.88	39.35	43.50	48.91	50.43
	pLF	78	26.68	28.38	32.40	35.75	38.55	39.85	42.11
	cMF	79	29.20	32.10	34.40	37.90	41.10	44.50	48.80
	pMF	79	31.80	33.50	36.90	40.00	44.50	48.10	51.30
	P	80	20.81	22.12	25.50	28.55	30.55	33.47	35.29
	LT	80	20.51	21.12	22.60	24.80	26.83	29.20	30.49
	MT	79	23.40	24.20	25.90	27.50	29.60	31.80	33.40
Female	cLF	63	33.36	35.42	39.20	43.60	49.20	52.84	54.22
	pLF	61	23.96	26.54	30.50	34.60	37.30	40.54	43.58
	cMF	63	26.82	30.06	35.70	39.60	43.50	47.82	49.38
	pMF	63	27.46	30.50	37.20	43.10	47.80	50.06	52.32
	P	63	18.48	22.38	24.60	27.90	31.50	34.50	37.48
	LT	63	19.90	20.80	22.70	25.70	29.50	33.52	36.66
	MT	63	22.82	23.56	26.40	29.20	31.10	34.40	36.50
**Age group 2 (42–50 years)**									
Male	cLF	67	31.48	33.02	36.40	42.10	48.30	53.52	56.20
	pLF	65	29.33	31.66	33.15	36.60	41.05	45.44	49.44
	cMF	63	27.94	31.32	35.70	40.30	45.30	50.30	51.60
	pMF	63	34.68	36.90	38.60	41.10	45.80	50.40	54.04
	P	68	23.69	25.39	26.60	30.50	34.70	37.32	38.60
	LT	67	21.00	22.34	23.70	26.10	28.70	31.94	33.84
	MT	67	22.12	23.18	25.10	29.00	32.00	34.84	35.26
Female	cLF	73	34.69	36.04	41.50	46.10	50.25	54.10	57.10
	pLF	73	25.10	26.60	29.90	33.60	37.80	38.80	40.82
	cMF	76	31.17	32.90	36.08	39.45	43.65	47.33	49.04
	pMF	76	32.96	34.47	38.45	43.50	747.70	51.80	53.05
	P	76	21.29	22.06	24.55	28.00	31.80	35.25	36.28
	LT	75	20.88	21.98	24.00	26.80	29.10	32.28	33.64
	MT	76	22.36	24.78	26.80	28.95	32.35	34.30	35.43
**Age group 3 (51–60 years)**									
Male	cLF	70	33.61	34.26	37.05	41.15	45.50	50.27	52.26
	pLF	69	28.65	30.40	33.35	38.20	42.25	45.00	45.60
	cMF	70	29.01	30.28	35.93	40.95	47.53	51.23	52.62
	pMF	70	35.07	36.63	40.40	44.45	47.48	50.40	52.65
	P	71	20.92	22.84	26.10	29.10	32.40	34.96	37.50
	LT	71	20.68	21.18	23.00	25.40	27.70	31.76	33.20
	MT	71	22.36	23.60	25.80	28.20	31.00	34.28	34.82
Female	cLF	66	34.38	35.98	39.40	42.50	47.20	50.56	53.51
	pLF	65	25.55	27.76	31.05	34.90	38.20	40.14	41.05
	cMF	70	30.71	32.81	34.73	41.10	44.43	48.07	50.10
	pMF	70	36.31	37.34	41.95	45.00	48.63	52.50	55.28
	P	70	18.26	19.68	23.10	27.15	31.50	36.07	37.21
	LT	69	17.90	20.10	22.00	25.40	28.55	30.40	32.50
	MT	68	21.20	22.69	24.80	28.45	31.80	33.16	35.42
**Age group 4 (61–85 years)**									
Male	cLF	83	32.80	35.10	39.30	43.40	47.60	51.90	55.24
	pLF	83	29.22	30.38	34.30	39.10	42.50	44.70	46.66
	cMF	76	29.11	30.14	32.70	40.35	44.20	50.15	53.03
	pMF	75	36.28	37.36	41.60	44.20	47.90	52.24	55.54
	P	83	20.84	21.92	24.40	28.00	32.60	39.62	40.48
	LT	83	19.28	20.44	22.90	24.70	27.90	29.92	32.18
	MT	82	21.12	21.83	23.50	26.60	29.33	31.40	34.35
Female	cLF	75	31.82	37.24	41.20	46.70	51.30	55.82	59.22
	pLF	74	28.33	30.85	33.50	37.15	41.75	46.50	49.25
	cMF	76	30.26	33.70	38.30	43.45	48.25	52.68	54.12
	pMF	75	32.28	35.56	41.00	46.70	50.90	55.74	56.92
	P	76	19.87	22.06	24.28	27.55	31.48	34.06	35.75
	LT	77	18.07	19.98	22.45	24.70	27.65	32.80	35.23
	MT	76	21.00	22.18	25.45	27.70	30.70	33.06	35.32

*cLF/pLF* central/posterior lateral femoral condyle, *cMF/pMF* central/posterior medial femoral condyle, *P* retropatellar cartilage, *LT* lateral tibia, *MT* medial tibia

### Sex-related differences in cartilage T2-times

**Fig. 5:**
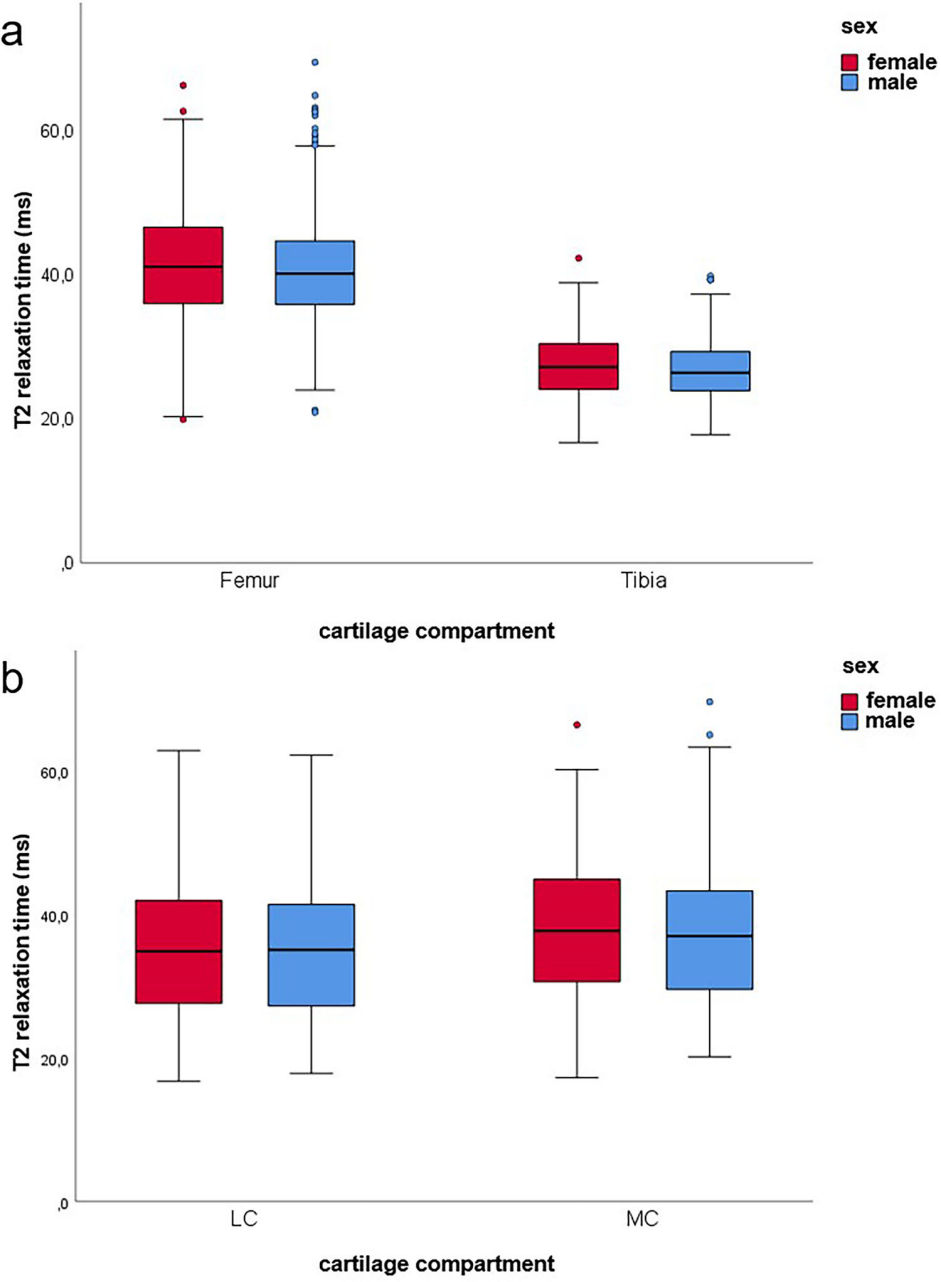
Illustration of the T2-relaxation time of the knee joint cartilage of women and men (with normal knee cartilage morphology) in comparison, divided according to additionally calculated compartments. **a** T2-relaxation times of the femoral and tibial cartilage. **b** T2-relaxation times of the lateral and medial compartments

Women had slightly higher T2-times than men in the entire knee cartilage, but the differences were not statistically significant overall. However, specific regions like cLF whole, cLF deep, and pMF superficial showed significant sex differences after adjusting for multiple testing. T2-times were significantly higher for women in all additionally calculated compartments (Fig. 5). Significant differences were observed in the femur (t(2229.366) = 2.352; *p* = 0.019), tibia (t(1126.036) = 2.470; *p* = 0.014), and medial compartment (t(1736) = 2.308; *p* = 0.021).

### Age-related differences in cartilage T2-times

Mean T2-times increased with age, with the oldest group (61–85 years) having the highest mean T2-time (35.71 ms, SD = 9.94 ms) and the youngest group (28–41 years) having the lowest (34.07 ms, SD = 8.13 ms). Age significantly influenced T2-times for the entire knee cartilage (F(3,2238.657) = 6.998, *p* < 0.001, ηp2 = 0.005), and for both superficial (F(3,2238.878) = 10.717, *p* < 0.001, ηp2 = 0.007) and deep layers (F(3,2238.143) = 2.751, *p* = 0.041, ηp2 = 0.002). Significant differences were only observed for the superficial layer, with the youngest group showing lower T2-times than older groups.

**Fig. 6:**
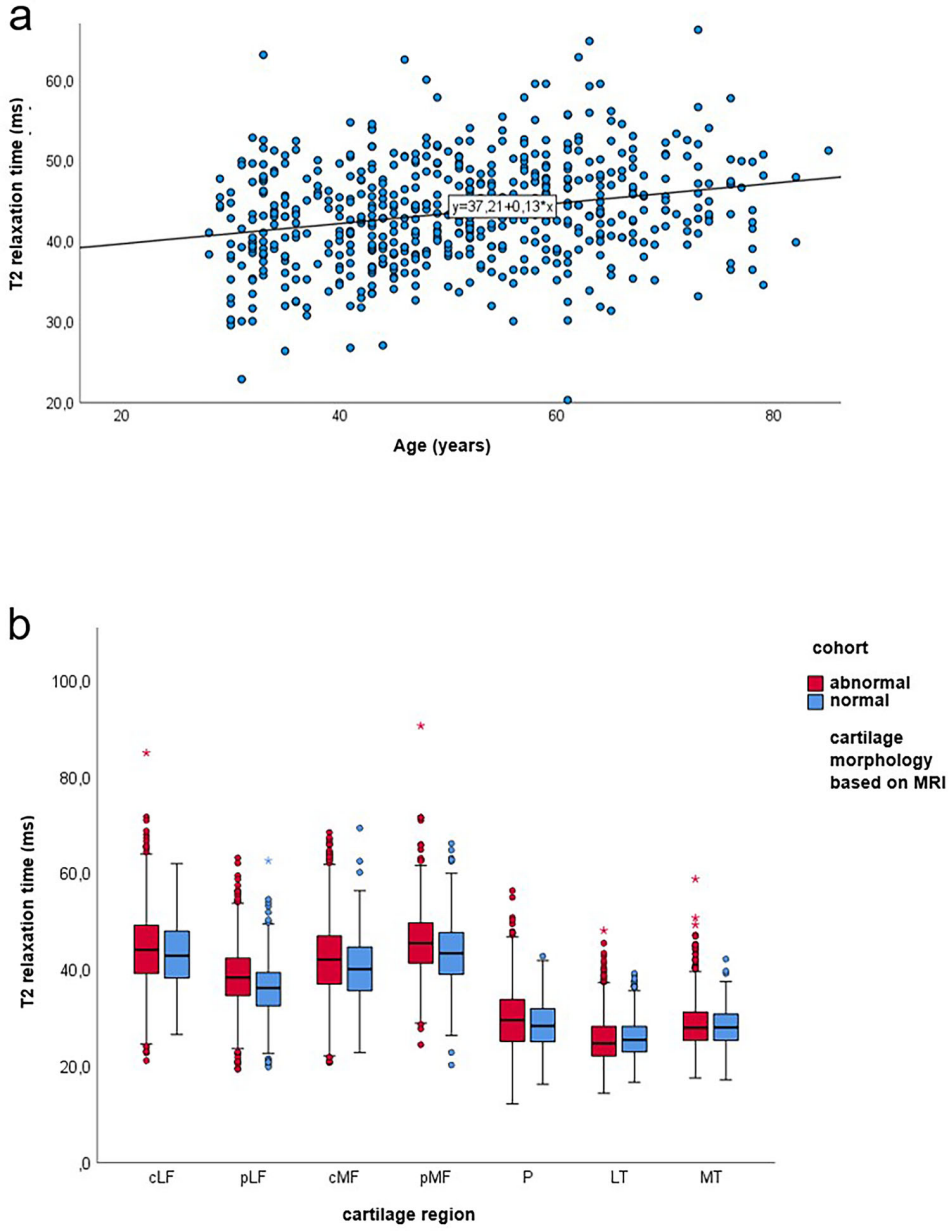
Assisiation between age and T2-relaxation time in cartilage and comparison of T2-values by cartilage morphology. **a** A significant, positive linear correlation between T2-relaxation time and age was demonstrated in the cartilage region cMF for subjects with normal knee cartilage morphology. **b** T2-relaxation times of the subcohorts (morphological normal versus abnormal knee cartilage), divided by cartilage region

Pearson analysis showed a positive correlation between age and T2-time (r = 0.060, *p* < 0.001). Regression analysis confirmed a positive linear relationship, indicating a 0.42 ms increase in T2-time per decade (F(1, 4039) = 14.742, *p* < 0.001). Figure 6a shows a scatter diagram that illustrates the linear relationship between the measured T2-time in the cartilage region cMF and age. This correlation was significant for all femur regions but not for the P and LT regions. The MT region showed a significant negative correlation, with a decrease of 0.4 ms per decade.

### Differences between subjects with morphological normal cartilage and subjects with morphological changes in knee cartilage based on MRI

The cohort with pathologic changes had significantly higher T2-values in all cartilage layers compared to the cohort with structurally normal knee cartilages (total whole: t(8955.193) = 7.541, *p* < 0.001; total superficial: t(9074.378) = 7.573, *p* < 0.001; total deep: t(8726.860) = 6.601, *p* < 0.001). T2-times were 1–2 ms higher in regions cLF, pLF, cMF, pMF, and P. Regions LT and MT showed no significant differences (Fig. 6b).

### Evaluation of multiple factors influencing knee cartilage T2-time

A mixed linear regression analysis assessed the influence of sex, age, cartilage region, and cohort type (MRI normal versus pathological altered knee cartilage) on T2-time. The analysis revealed that cartilage region and cohort type significantly influenced T2-time (*p* < 0.001), while sex (*p* = 0.369) and age (*p* = 0.133) did not. The cartilage region had the most significant impact on T2-time. Detailed results of the regression analysis can be found in Table 5.

**Table 5:** Estimates of fixed parameters of the mixed linear regression

Parameter	Estimator	Std. error	df	T	*p*-value	95% CI	
						Lower limit	Upper limit
Constant term	28.18	0.60	987.30	46.90	< 0.001	27.00	29.36
Male	−0.21	0.23	910.54	−0.90	0.369	−0.65	0.24
Female	0.00^a^	0.00	.	.	.	.	.
Age	0.01	0.01	917.17	1.50	0.133	0.00	0.03
cLF	15.69	0.18	11,445.74	86.94	< 0.001	15.34	16.04
pLF	9.45	0.18	11,449.81	52.23	< 0.001	9.10	9.81
cMF	13.32	0.18	11,444.61	73.31	< 0.001	12.97	13.68
pMF	16.63	0.18	11,443.82	91.86	< 0.001	16.27	16.98
P	0.91	0.18	11,444.97	5.04	< 0.001	0.56	1.27
LT	−2.83	0.18	11,443.63	−15.72	< 0.001	−3.18	−2.48
MT	0.00^a^	0.00	.	.	.	.	.
Normal	−1.24	0.26	908.99	−4.85	< 0.001	−1.74	−0.74
Abnormal cartilage morphology	0.00^a^	0.00	.	.	.	.	.

Dependent variable: Mean T2 relaxation time (in ms) of the entire knee joint cartilage; Fixed effects: sex, age, region, subcohort; Random effects: Subject ID

*cLF/pLF* central/posterior lateral femoral condyle, *cMF/pMF* central/posterior medial femoral condyle, *P* retropatellar cartilage, *LT* lateral tibia, *MT* medial tibia

Age (in years)

^a^this parameter is set to zero as it is redundant

## Discussion

### Cartilage region-dependent variability in T2-relaxation times

This study investigated the T2-relaxation time of knee joint cartilage in subjects from the SHIP-TREND-1 cohort, evaluating morphological abnormalities of the knee joint cartilage and the influence of age, sex, and cartilage region on T2-relaxation times. T2-reference values for the cartilage of subjects with normal MRI morphology were also calculated. This research presents the 1.5 Tesla T2-relaxation times of knee joint cartilage in the largest group to date (929 subjects, 1824 knee joints).

This study demonstrates that the cartilage region significantly influenced T2-time in all cartilage layers. On average, the femoral cartilage had the highest T2-time, followed by the retropatellar cartilage, with the tibial cartilage having the lowest T2-time. These findings align with Joseph et al who also reported higher T2-values in femoral cartilage and lower T2-values in patellar and tibial cartilage [11]. The superficial cartilage layer consistently showed higher values than the deep layer, reflecting the biochemical structure of cartilage with higher proteoglycan and lower water content in deeper layers [38–40]. The medial joint compartment exhibited significantly higher T2-values than the lateral compartment, as seen before [11, 12, 41], likely due to greater stress during walking and a higher incidence of osteoarthritis [42, 43].

The calculated T2-reference values of this study suggest that T2-values vary with demographic characteristics, an important consideration when examining T2-values in OA context. Although reference values may not clearly differentiate between ‘normal’ cartilage tissue and early stages of degeneration, studying morphologically healthy cartilage tissue T2-values by demographic factors enhances understanding and interpretation of these values.

### Demographic influences on T2-values: age and sex effects

The calculated normal T2-values of this study suggest that T2-values vary with demographic characteristics, an important consideration when examining those in OA context. Although T2-mapping may not clearly differentiate between biologically healthy cartilage tissue and early stages of degeneration, studying morphologically healthy cartilage tissue T2-values by demographic factors enhances understanding and interpretation of these values.

Sex differences in T2-times were observed, with women having higher T2-times than men across the entire knee joint cartilage in all layers, though these differences were not statistically significant. In individual cartilage regions, T2-time was significantly higher in women: in the femoral and tibial cartilage and the medial compartment. Our results are partially consistent with prior studies that also reported higher T2-time for women in subregions of the tibia and femur [11, 12]. Mosher et al examined a comparatively young population (22–29 years) and were unable to calculate a significant influence of sex on T2-time [16]. These higher T2-values in women may either indicate a sex-related physiological difference in cartilage composition [44] or early degenerative cartilage changes, consistent with epidemiological studies showing a higher prevalence of osteoarthritis (OA) in women [23].

Age significantly influenced cartilage T2-time. The youngest age group had significantly lower T2-times than all other age groups. However, significant differences were found only in the superficial cartilage layer, suggesting that age-related cartilage changes occur primarily in this layer, as previously reported [15, 16]. A positive linear correlation between age and T2-time was also identified, echoing results from Joseph et al [11].

### Association of elevated T2-values with morphological cartilage changes

The presence of morphological changes in knee cartilage significantly influenced T2-relaxation time, with the cohort with structural cartilage lesions showing higher T2-values across all layers than the cohort with normal knee cartilage based on MRI, corroborating findings by Dunn et al and Joseph et al [45, 46]. The cohort with morphological changes in knee cartilage also demonstrated greater variance in T2-times. The results of our study are additionally supported by the findings of other studies, which have already shown that the T2-relaxation time of cartilage is sensitive to processes of early cartilage degeneration, such as changes in water content [47] and water mobility [16], as well as the alignment of collagen fibers [48].

### Multivariate influences on T2-relaxation times

A mixed linear regression analysis showed that cartilage region and cohort (normal versus abnormal cartilage morphology) significantly affected T2-time. Age and sex had significant region-specific influences. These findings are consistent with previous studies [11, 16, 27]. This study demonstrated that T2-mapping and cartilage morphology assessment using the modified Noyes score are reliable for assessing cartilage morphology and composition in large populations. The results suggest that T2-mapping could serve as a non-invasive biomarker for early OA diagnosis, detecting natural variation in T2-time based on demographic factors and regional variations within cartilage compartments.

### Technical advantages and limitations of T2-mapping

T2 mapping is a widely available, non-invasive technique that allows for early detection of cartilage degeneration by reflecting changes in water content, collagen fiber orientation, and proteoglycan loss. However, limitations currently prevent routine clinical use of T2-values. These include difficulties in comparing T2-relaxation times across different studies and systems due to varying MR devices, sequences, and T2-map creation methods [49–52]. Standardizing image acquisition, phantom validation, post-processing, and cartilage segmentation is crucial to improving study comparability and T2-value assessment in longitudinal studies. In this study, a multi-slice multi-echo (MSME) sequence was used, which is commonly implemented across vendors and compatible with standard MRI systems. T2 maps were automatically generated using vendor-provided software based on mono-exponential fitting. Although more advanced fitting models (e.g., 3-parameter fitting) may offer improved accuracy, particularly in deep cartilage layers, they were not available within our processing environment and may be less feasible in large-scale clinical studies.

Our protocol did not include phantom-based validation, which limits the direct comparability of absolute T2-values across studies or scanners. At the time of data acquisition, a standardized approach like the QIBA reference protocol had not yet been widely established [53]. We acknowledge that the lack of harmonized acquisition and post-processing standards remains a key limitation in quantitative cartilage imaging and emphasize the need for standardized protocols and cross-site validation in future research.

Despite these limitations, the consistent methodology within this large, population-based cohort ensures internal validity and provides robust data for cartilage T2 relaxation at 1.5-T MRI.

### Definition of the ‘healthy cohort’ and confounding factors

Another limitation of this study is the definition of the ‘healthy cohort’. The definition is based solely on the evidence of morphological changes in the knee cartilage based on MRI. We did not investigate additional features such as other structural abnormalities, diseases, complaints and other factors that would favor the presence of degenerative changes in the knee joint. The T2-values for knee cartilage obviously depend on many different factors, such as genetic characteristics, environmental exposure, social factors such as lifestyle, education and health awareness, population mobility, average BMI and others. This has to be generally taken into account when interpreting the T2-values.

The substantial variance in measured values (mean T2-difference between raters of 2–3 ms), relative to the small T2-differences observed between the examined groups (1–2 ms between the two cohorts), also represents a limitation of this study. This variance could be reduced with more precise standardization of T2-mapping.

### Population representativeness

The SHIP-TREND-1 cohort utilized in this study is a well-characterized, population-based sample from north-eastern Germany, designed to reflect the demographic and health characteristics of the general adult population in the region as closely as possible.

The SHIP-TREND-1 cohort is predominantly composed of individuals of Caucasian descent, enhancing the applicability of our findings to similar populations in Central and Northern Europe. While regional differences in lifestyle, occupational exposure, and healthcare access may influence musculoskeletal health, the demographic and anthropometric similarities support the broader relevance of our results. Nevertheless, we acknowledge that generalization to non-European populations or regions with substantially different lifestyle factors should be approached with caution.

## Conclusion

This study provides normal T2 relaxation values for knee cartilage based on a large, population-based cohort examined with 1.5 T MRI, stratified by age, sex, and cartilage layer. The results confirm age- and sex-related differences in T2-values, as well as elevated T2 times in the presence of morphological cartilage degeneration.

Given the demographic and anthropometric characteristics of the SHIP-TREND cohort—including balanced sex distribution, a broad adult age range, and BMI values consistent with national and European averages—these reference values are likely generalizable to similar Western Caucasian populations. The cohort’s population-based design enhances external validity, supporting applicability to adult populations in Central and Northern Europe. However, generalization to non-European populations or regions with substantially different lifestyle and environmental conditions may be limited.

These normal T2-values may serve as a robust foundation for clinical assessment, medical education, and the development of AI-based diagnostic tools for early detection and monitoring of cartilage degeneration. T2 mapping remains a promising non-invasive imaging biomarker for early osteoarthritis, especially in the context of population-based screening and longitudinal studies.

## Supplementary information

ELECTRONIC SUPPLEMENTARY MATERIAL

## Abbreviations

cLF: Central (“weight bearing”) lateral femoral condyles
cMF: Central (“weight bearing”) medial femoral condyles
F: Femur
Fig: Figure
FOV: Field of view
Hz: Hertz
LC: Lateral compartment
LT: Lateral tibia
MC: Medial compartment
MSME: Multi-slice multi-echo sequence
MT: Medial tibia
OA: Osteoarthritis
P: Patella
pLF: Posterior lateral femoral condyles
pMF: Posterior medial femoral condyles
Px: Pixel
RMSA CV: Root mean square average coefficients of variation
ROI: Region of interest
SD: Standard deviation
SE: Spin-echo
SHIP: Study-of-health-in-Pomerania
T: Tibia
TE: Echo time
TR: Repetition time
