# Supplementary material for: Association of layer-specific knee cartilage T2-relaxation measurements with age, sex and cartilage morphology at 1.5-T MRI
Source: Eur Radiol. 2025 Jul 25;36(1):308–23. doi: 10.1007/s00330-025-11806-8 (PMC12711982; doi:10.1007/s00330-025-11806-8)
Supplement: Supplementary file 1 — ELECTRONIC SUPPLEMENTARY MATERIAL [file 330_2025_11806_MOESM1_ESM.pdf]

Association of layer-specific knee cartilage T2-relaxation measurements with age, sex and cartilage morphology at 1.5T MRI

ELECTRONIC SUPPLEMENTARY MATERIAL

Suppl. Fig.1: Illustration of ROI analysis in the lateral articular compartment: placement of ROI in the sagittal T2 MSME-SE sequence (A), then copying ROI to the T2-color map (B).

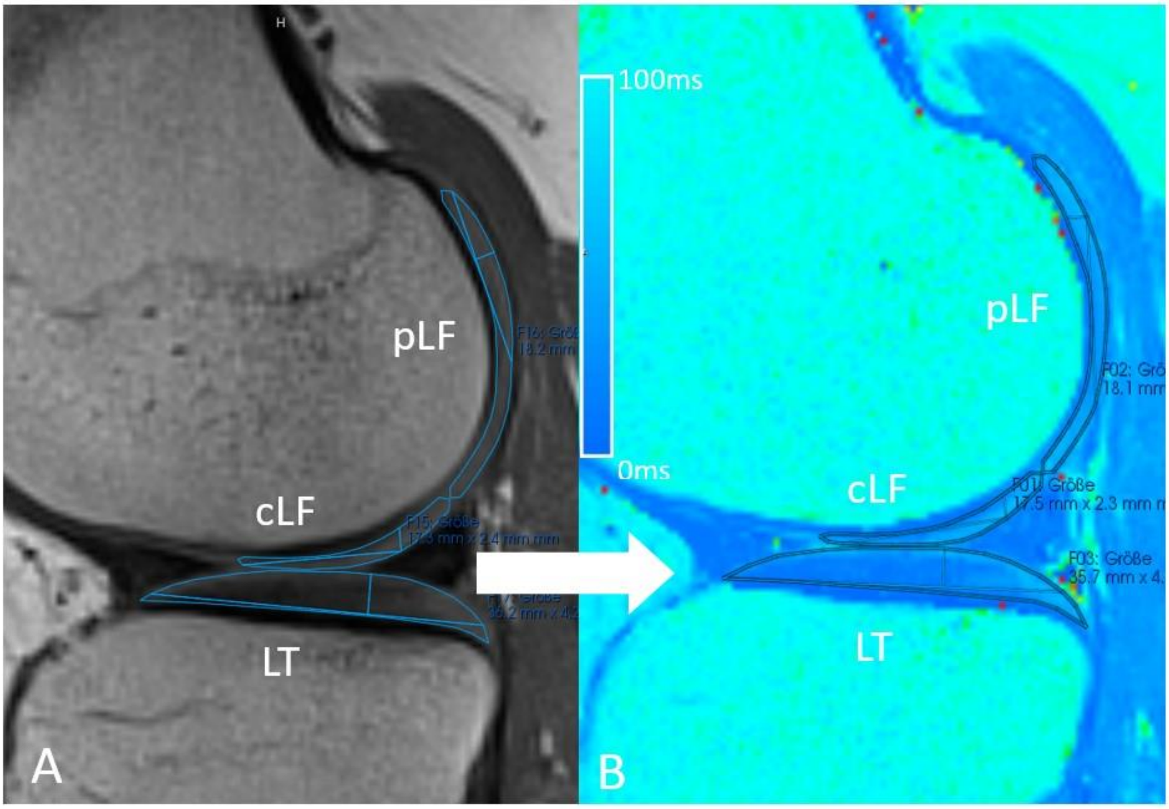

Source: study participant from the SHIP-TREND-1 data pool.

Suppl. Fig. 2: Modified Noyes grading in MRI

The coronal (A, B, C) and sagittal proton-weighted fat-saturated images show an internal signal change of the tibial articular cartilage (grade 1, A) and a cartilage defect <50 % of the total thickness (grade 2, B); a cartilage lesion >50 % of the total of the total thickness in the area of the femoral condyle (grade 3, C), a defect that extends to the subchondral bone (grade 4, D)

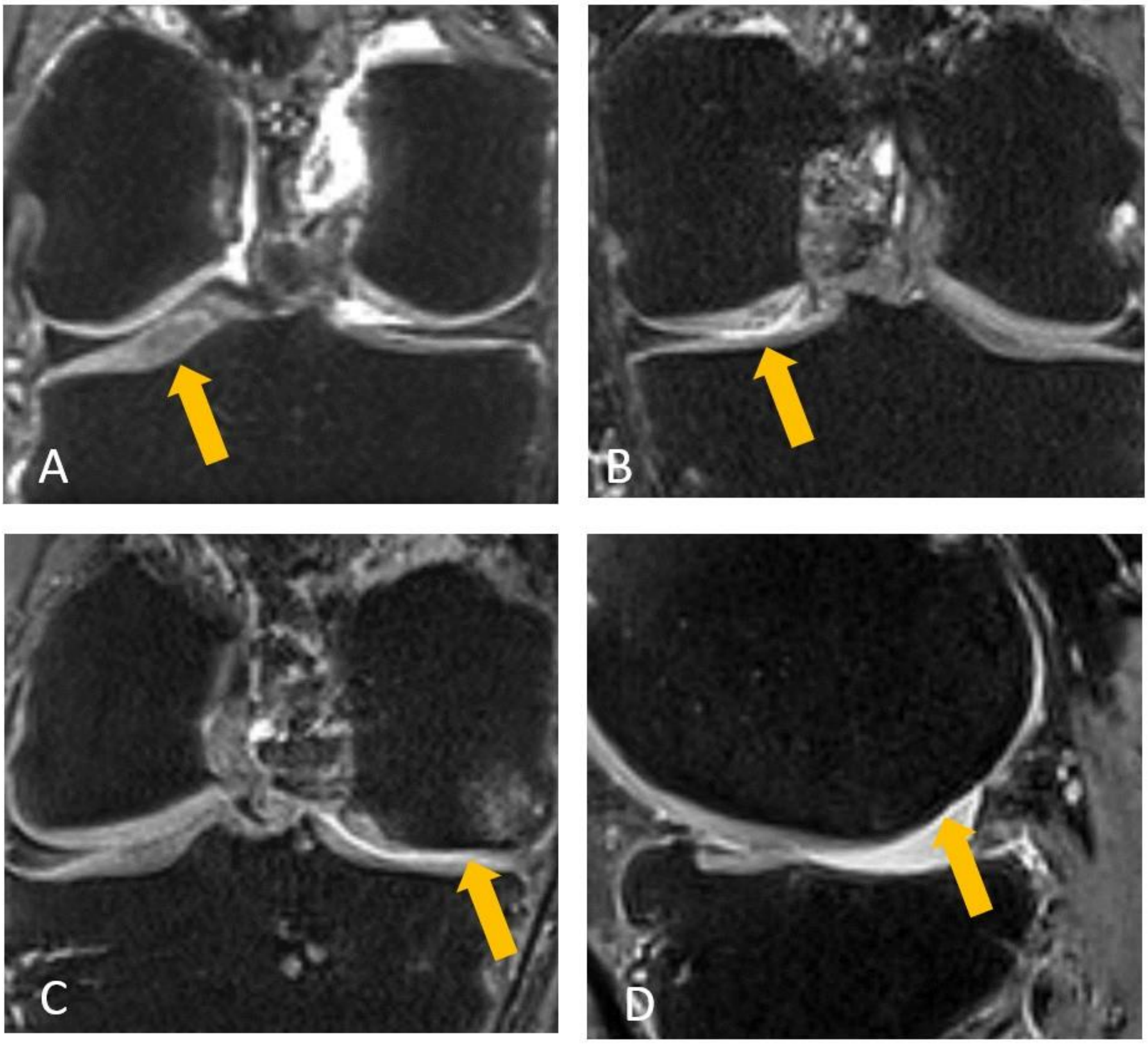

Source: study participant from the SHIP-TREND-1 data pool.

Supplementary Table: Parameters of the MRI sequences used.

	T1 SE axial	T1 SE coronar	3D PD fat-sat SPACE sagittal	T2 MSME-SE sagittal
TR (ms)	305	511	1200	1060
TE (ms)	11	11	30	13,8/27,6/41,4/ 55,2/69,0
field of view (mm)	160 * 160	160 * 160	180 * 180	160 * 160
matrix (pixel)	154 * 192	205 * 256	258 * 256	256 * 256
slice thickness (mm)	3,0	3,0	0,7	3,0
bandwidth (Hz/Pixel)	151	149	698	227
scanning time (min)	01:10	01:44	07:05	02:23
